# Supplementary figures and images for: Sedimentary DNA is a promising indicator of the abundance of marine benthos: Insights from the burrowing decapod Upogebia major
Source: PLoS One. 2025 Mar 19;20(3):e0318235. doi: 10.1371/journal.pone.0318235 (PMC11922209; doi:10.1371/journal.pone.0318235)

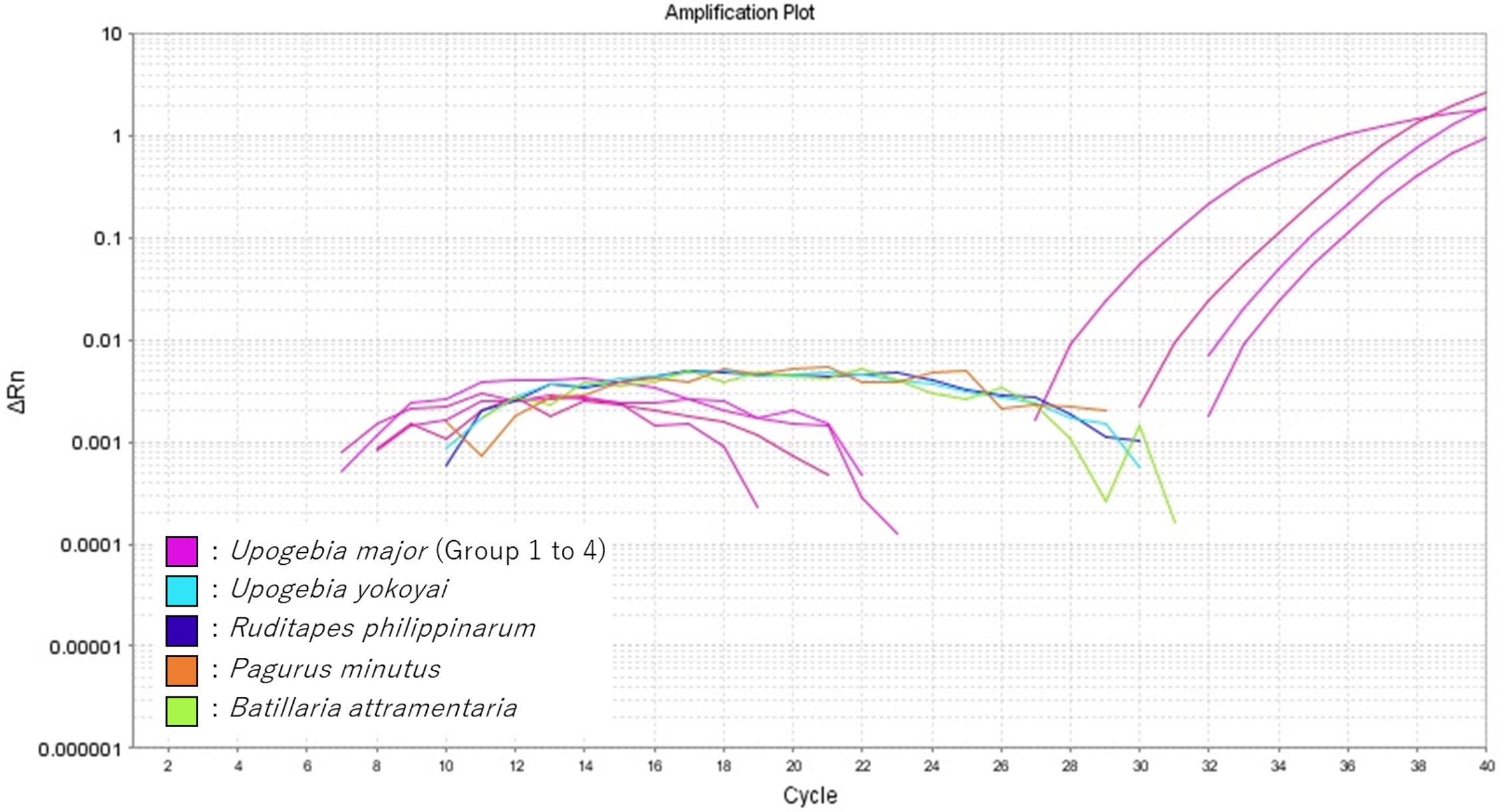

Supplement: S1 Fig — The organisms targeted for this assessment included U. major, its closely related species U. yokoyai, the dominant species at each survey site, R. philippinarum, P. minutus and B. cumingii. The number of samples is one for each species (one sample per group for U. major). The amplification was confirmed for only the four U. major samples from Groups 1 to 4. (JPG) [file pone.0318235.s001.jpg]
