# Supplementary material for: Sedimentary DNA is a promising indicator of the abundance of marine benthos: Insights from the burrowing decapod Upogebia major
Source: PLoS One. 2025 Mar 19;20(3):e0318235. doi: 10.1371/journal.pone.0318235 (PMC11922209; doi:10.1371/journal.pone.0318235)
Supplement: Attachment — Submitted filename: Response to Reviewers (Kitabatake et al. 2024).docx [file pone.0318235.s006.docx]

**Response to Reviewers**

Dear Dr. Luísa Borges:

Thank you for giving us to submit a revised draft of our manuscript entitled, “Sedimentary DNA is a promising indicator of the abundance of marine benthos: Insights from the burrowing decapod *Upogebia major*” to PLOS ONE.

We greatly appreciate the time and effort you and the reviewers have dedicated to providing insightful feedback to strengthen our paper. It is with great pleasure that we resubmit our article for further consideration. We have incorporated changes that reflect the detailed suggestions you graciously provided. We hope that our edits and the responses provided below satisfactorily address all the issues and concerns noted by you and the reviewers.

Below, we provide responses to the comments we received.

**Reviewers' Comments to Author:**

**Reviewer 1**

The MS seems likely to satisfy all of the criteria for publication in PLOS if it can be significantly revised. The article is written in excellent, standard English and presents the results of original research that has not been published elsewhere. The research also appears to meet all applicable standards for the ethics of experimentation and research integrity and the article adheres to appropriate reporting guidelines and community standards for data availability.

REPLY: Thank you very much for taking the time to review our manuscript despite your busy schedule. We sincerely appreciate your thorough and meticulous review of our manuscript. Your comments have been invaluable in improving the quality of our paper.

The experiments, statistics, and other analyses, however. are not performed to a high technical standard or described in sufficient detail. Kitabatake, for example, mention one period where detections were lower in non-shrimp areas than in abundant shrimp areas but they detected sedDNA nearly everywhere. They do not provide a simple graph of sedDNA detections and burrow or shrimp abundance to indicate an overall correspondence with abundance. (Wouldn’t the expected correlation be better with biomass?) Their conclusions therefore do not appear to be presented in an appropriate fashion to demonstrate they are supported by their data.

REPLY: Thank you very much for your valuable feedback. As *U. major* is not easy to collect, it was challenging to directly compare abundance (biomass) with sedDNA concentration. Therefore, in this study, we conducted the sedDNA analysis based on the assumption that "number of burrow openings ≈ abundance." We have added this explanation to Lines 128–141.

It is not clear to this reviewer how “The findings of this study”. “demonstrate the effectiveness and optimal conditions of sedDNA analysis for estimating the abundance of marine benthos” and also “also suggested new potential in sedDNA for evaluating the physiological activity of benthos.”

Presumably, sedDNA is a function of production relative to degradation but they don’t they say that. These problems should be easy to fix. Kitabatake et al., for instance, could mention that better estimates DNA degradation (with distance [samples at greater distances from the shrimp beds] and time [repeated samples of the same sediments containing *U. major* DNA]) are still needed for determining abundance.

REPLY: Thank you very much for your invaluable advice. As you rightly pointed out, evaluating the dynamics of sedDNA degradation is a crucial aspect when using sedDNA concentration as an indicator of abundance. We have addressed this point by adding further details in Lines 674–680.

The burrow opening size/morphology information seems to be a different story and largely irrelevant. Except as estimates of abundance, those data seem to distract from the sedDNA detection story. Their explanation of how the burrow diameter or type is, or could be, related to sedDNA detection is confusing. Burrow diameter times burrow number could be crudely related to biomass but Kitabatake don’t use those data for that. Sediments from their tank experiment, described briefly, was not sampled for sedDNA. Samples were not held and tested over time to test for degradation of reductions of detection anywhere.

REPLY: Thank you very much for your valuable feedback. We believe that the morphology and diameter size of burrow openings are related to sedDNA detection. Type 1 burrows are frequently observed during periods of high physiological activity, such as spawning and growth periods, and these periods correspond to higher sedDNA detection rates. Additionally, the larger diameter of Type 1 burrows likely facilitates the release of more eDNA from within the burrows to the external environment. In contrast, periods when Type 2 or Type 3 burrows are more prevalent coincide with winter or high-temperature seasons, during which physiological activity is expected to be lower. Furthermore, the smaller diameter of these burrows likely restricts the release of sedDNA produced inside the burrows to the external environment. We have added a discussion on the significance of burrow morphology and diameter size in Lines 527–533.

Regarding the estimation of biomass by multiplying the diameter and number of burrow openings: At least for Type 1 and Type 2 burrows, their formation is not necessarily related to the growth stage, and there is no consistent correlation between the diameter of the openings and the body size of the individuals. Specifically, for Type 2 burrows, even large individuals with a carapace length of approximately 30 mm often create openings with diameters of less than 1 cm (please refer to Fig 3). Additionally, as described in Lines 389–391, an increase in Type 2 and Type 3 burrows makes it more difficult to locate the positions of the burrows, leading to a decrease in the apparent number of burrows. For these reasons, estimating biomass based on the diameter and number of burrow openings carries concerns regarding accuracy, and we did not perform such an estimation in this study.

Next, regarding the tank experiment, this study focused on investigating the relationship between the morphology of burrow openings and the behavior of *U. major* within the burrows, and therefore, sedDNA analysis was not conducted. Additionally, the description of the tank experiment has been revised, with the information now separated into the Methods and Results sections. Please refer to Lines 200-210 and 407–417 for further details.

Finally, regarding the experiment to investigate the degradation of sedDNA, as mentioned in Lines 674–680, this is considered a future challenge. This study represents the first step toward developing a method for estimating abundance from sedDNA concentrations. To achieve this goal, we believe it is essential to first evaluate the overall relationship between *U. major* abundance and sedDNA concentrations, and this study focused on that aspect. Building on the results of this study, we plan to conduct further research in the next step, focusing on the dynamics of sedDNA, which will contribute to the accurate interpretation of sedDNA concentration data.

Line 87-88: “in recent years, the quantitative assessment of this species has decreased.” What does this mean? Is there a reference?

REPLY: Thank you for your comment. The relevant part refers to the fact that quantitative surveys have rarely been conducted in recent years. Since the original phrasing was potentially misleading, we have revised the sentence accordingly (Lines 89–90).

Line 121: “*U. major* is a generalist” – this requires an explanation.

phrasing REPLY: We have revised the expression in the relevant section. We would appreciate it if you could review Lines 122–123.

Line 226: sampled with - “sterilized plastic spoons soaked in 70% ethanol”. Ethanol preserves DNA.

Explain how this avoided sample contamination. Was a different sterilized plastic spoon used for each sample?

REPLY: The term 'ethanol' was a mistake, and it should have been 'chlorine bleach,' so I have made the correction. Additionally, a different sterilized spoon was used for each sample, and the spoons were not reused. Please refer to Lines 251–252 for the revision.

**Reviewer 2:**

This paper is a challenging attempt to use sedDNA as an indicator to examine the density of a large uopogebiid shrimp, which is an ecosystem engineer in tidal flats of the West Pacific. The results are not easy to interpret, as they are influenced by a number of factors, including the shape of the tidal flat, seasonal changes in the activity of the shrimp, and the dispersion and degradation rates of sedDNA. Even so, the authors have given honest and accurate consideration to each result, and there are no parts where the logic breaks down. Because the data was collected appropriately, the data published in this paper is expected to gradually increase in value as future research develops.

REPLY: Thank you very much for taking the time to review our manuscript despite your busy schedule. We sincerely appreciate the additional information provided, which has allowed for a more in-depth discussion of the results of our study. The valuable comments you have given have been immensely helpful in revising the paper.

Please refer to the following on the method of displaying and analyzing the results.

L. 284. The statistical analysis methods should be in a separate section. It is also not good to have the details written as captions for supplementary tables. Since the presence or absence of seasonal and spatial changes is mentioned in the main text, please describe the analysis methods in the main text.

REPLY: Thank you for your comment. I have separated the statistical analysis methods into a different section and included them in Lines 309–325.

Perhaps Figures 5 and 6 are the most important results, but you need to explain in the Methods section what you are comparing there, and you also need to make the figures of the results easy to understand.

In particular, when it comes to seasonal changes, it is only appropriate to compare the burrow-rich area, and this should be shown in a bar graph like Figure 6. When I only look at Table S7, it looks as if there are no seasonal changes.

REPLY: Thank you very much for your valuable feedback. The details of the comparisons conducted in this study (1: Comparison of sedDNA concentrations between burrow-rich areas and areas without burrows within the same tidal flat. 2: Comparison of the number of burrows and sedDNA concentrations in tidal flats without distinct burrow-rich areas, where burrows are distributed throughout the entire flat) and the criteria for determining the correlation between abundance and sedDNA concentrations have been added at the end of the introduction. We would appreciate it if you could review Lines 126–141. Additionally, we have added a bar graph showing the seasonal variation in sedDNA concentrations within the burrow-rich area. We would appreciate it if you could kindly check Figure 7b.

By the way, isn't the order of the letters of transect ABCD in Figure 2b wrong?

I think it doesn't match up with Figure 5.

REPLY: Revised.

L. 409. There is no description in the Methods section about the behavioral observation. If you are going to describe the results, you need to describe the methods. However, even if these behaviors or activities were not observed at low tide, it is not possible to deny the possibility that they are carried out at high tide, so I think that this is an extremely limited observation that supports the authors' discussion.

The description from L. 513 to L. 526 cannot be used in the discussion unless it is described in the methods and results. I think it would be good to summarize the methods and observation in the supporting information.

REPLY: We have separated the description of the tank experiment into the Methods and Results sections. Please refer to Lines 200–210 and 407–417. Additionally, we have included details regarding *U. major*'s burrow formation behavior during low and high tides.

The following are important reviews of the ecology of the upogebiid shrimps, so please add them to your citations.

Atkinson, R. J. A. and A. C. Taylor (2005). "Aspects of the physiology, biology, and ecology of thalassinidean shrimps in relation to their burrow environment." Oceanography and Marine Biology: An Annual Review 43: 173-210.

Kinoshita, K. (2022) “Life history characteristics and burrow structure of the mud shrimp (Decapoda: Upogebiidae)” Plankton and Benthos Research 17: 327-337/

REPLY: Thank you very much for sharing the reference. We have added Atkinson & Taylor (2005) to the list of references; please refer to Line 651. As for Kinoshita (2022), it has already been cited (see Line 727).
